# Genotoxic properties of XLR-11, a widely consumed synthetic cannabinoid, and of the benzoyl indole RCS-4

**DOI:** 10.1007/s00204-016-1664-4

**Published:** 2016-02-08

**Authors:** Franziska Ferk, Richard Gminski, Halh Al-Serori, Miroslav Mišík, Armen Nersesyan, Verena J. Koller, Verena Angerer, Volker Auwärter, Tao Tang, Ali Talib Arif, Siegfried Knasmüller

**Affiliations:** 1 Department of Internal Medicine 1, Institute of Cancer Research, Medical University of Vienna, Borschkegasse 8A, 1090 Vienna, Austria; 2 Environmental Health Sciences and Hospital Infection Control, Medical Center, University of Freiburg, 79106 Freiburg, Germany; 3 Institute of Forensic Medicine, Medical Center, University of Freiburg, 79104 Freiburg, Germany; 4 Institute of Earth and Environmental Science – Geochemistry, University of Freiburg, 79104 Freiburg, Germany

**Keywords:** Synthetic cannabinoids, Genotoxicity, SCGE assay, Micronucleus assay

## Abstract

Aim of this study was the investigation of the genotoxic properties of XLR-11 [1-(5-fluoropentyl)-1*H*-indol-3-yl](2,2,3,3-tetramethylcyclopropyl)methanone, a widely consumed synthetic cannabinoid (SC), and of the benzoyl indole RCS-4 (4-methoxyphenyl)(1-pentyl-1*H*-indol-3-yl)methanone). We characterized the DNA-damaging properties of these drugs in different experimental systems. No evidence for induction of gene mutations was detected in bacterial (*Salmonella*/microsome) tests, but clear dose-dependent effects were found in in vitro single cell gel electrophoresis (SCGE) assays with human lymphocytes and with buccal- and lung-derived human cell lines (TR-146 and A-549). These experiments are based on the determination of DNA migration in an electric field and enable the detection of single- and double-strand breaks and apurinic sites. Furthermore, we found that both drugs induce micronuclei which are formed as a consequence of chromosomal aberrations. The lack of effects in SCGE experiments with lesion-specific enzymes (FPG, Endo III) shows that the DNA damage is not caused by formation of oxidatively damaged bases; experiments with liver enzyme homogenates and bovine serum albumin indicate that the drugs are not converted enzymatically to DNA-reactive intermediates. Furthermore, results with buccal- and lung-derived human cells show that gaseous treatment of the cells under conditions which reflect the exposure situation in drug users may cause damage of the genetic material in epithelia of the respiratory tract. Since DNA instability is involved in the etiology of cancer, these findings can be taken as an indication that consumption of the SCs may cause tumors in the respiratory tract of consumers.

## Introduction

Synthetic cannabinoids (SCs) are drugs which bind to the cannabinoid receptors CB_1_ and CB_2._ They cause similar psychotropic effects as Δ^9^-tetrahydrocannabinol (Δ^9^-THC). These compounds are marketed worldwide in form of herbal mixtures (“legal highs”) as a surrogate for marijuana (Spaderna et al. 2013).

The toxic properties of these drugs are in general only poorly investigated. We found in a recent investigation with human-derived cells that some of them cause damage of the genetic material, while other toxic effects (acute cytotoxicity, immunological effects and estrogenic properties) were only moderate or lacking (Koller et al. 2013, 2014). Genetic damage is involved in etiology of various diseases, i.e., it plays a key role in the induction of cancer and is also involved in neurodegenerative disorders, atherosclerosis, infertility and aging (Ames and Gold 1997; Ames et al. 1993). Therefore, our previous findings indicate that adverse effects, which are related to DNA damage, may be induced in users of these drugs.

**Fig. 1:**
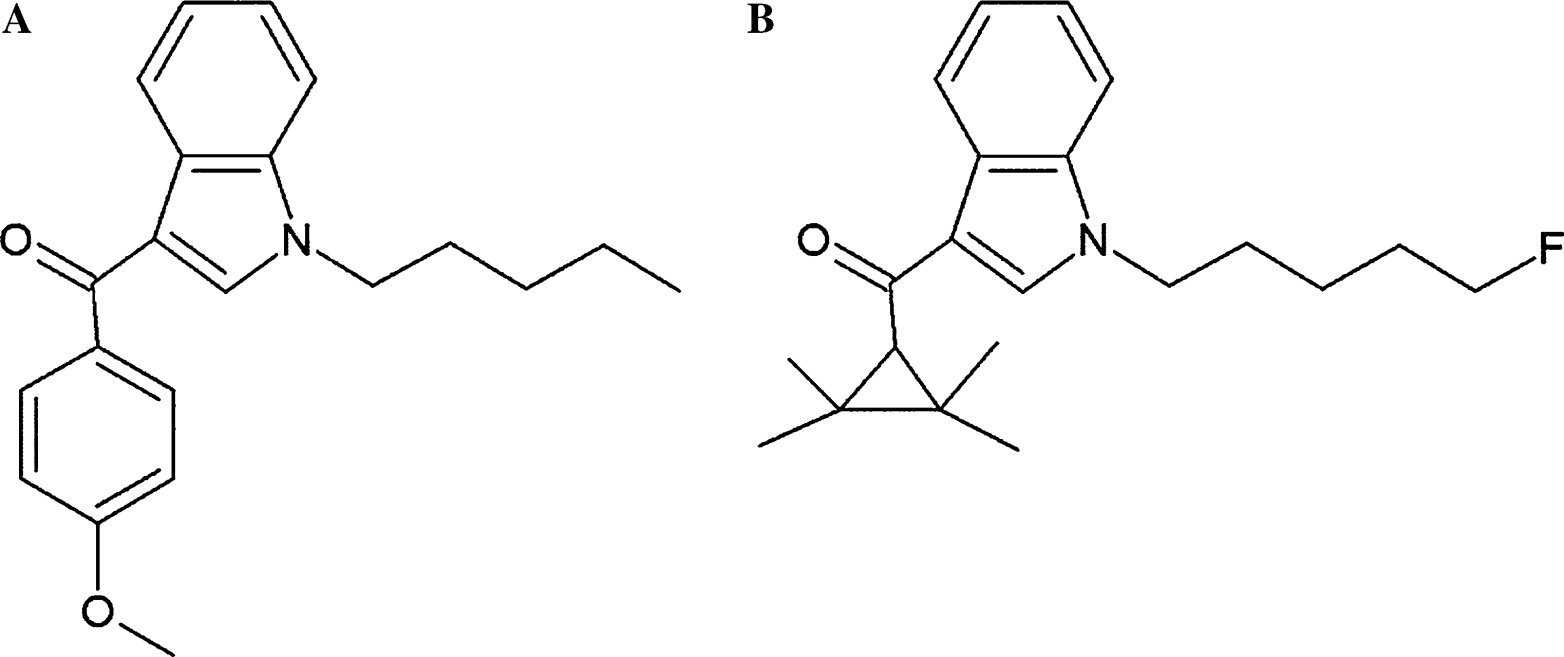
Chemical structures of the test compounds. **a** RCS-4 (4-methoxyphenyl)(1-pentyl-1*H*-indol-3-yl)methanone, CAS 1345966-78-0), **b** XLR-11 ([1-(5-fluoropentyl)-1*H*-indol-3-yl](2,2,3,3-tetramethylcyclopropyl)methanone, CAS 1364933-54-9)

Aim of the present study was the characterization of the genotoxic properties of XLR-11, a tetramethylcyclopropyl indole drug. This compound was one of the most widely used SCs in the USA and in the Europe and replaced several other drugs such as JWH-018 and AM-2201 (Uchiyama et al. 2013). It was named after the first fuel rocket developed in the USA and binds to both cannabinoid receptors (Wiley et al. 2013). Furthermore, we included in the present investigation also experiments with RCS-4, an indole-based SC which contains a benzoyl ring. It is structurally related to other drugs such as AM-694 and RCS-8 (Logan et al. 2012) and less frequently consumed than XLR-11 (Seely et al. 2013). Figure 1a, b depicts the chemical structures of the two compounds.

We used different experimental models to characterize the genotoxic properties of these drugs including bacterial mutagenicity tests with *Salmonella typhimurium* strains which detect gene mutations (Maron and Ames 1983) and single cell gel electrophoresis (SCGE or “comet”) assays which are based on the measurement of migration of DNA in an electric field and detect single- and double-strand DNA breaks and apurinic sites (Tice et al. 2000). These experiments were conducted with the buccal-derived human cell line TR146 (Rupniak et al. 1985) and additionally with peripheral human lymphocytes. To determine whether the drugs are converted by phase I enzymes to DNA-reactive intermediates, further SCGE experiments were conducted with liver enzyme homogenate and bovine serum albumin. The formation of oxidatively damaged DNA was assessed in comet experiments by use of lesion-specific enzymes (formamidopyrimidine DNA glycosylase and endonuclease III).

Since “comets” disappear as a consequence of repair processes, it is not clear whether they cause persisting alterations of the genetic material. To clarify this question, we studied in subsequent experiments the induction of micronuclei (MNi) which are formed as a consequence of structural and numerical chromosomal aberrations and of other nuclear anomalies in cytome assays (Fenech 2007).

Finally, experiments were conducted with an Air–liquid interface exposure system which allows investigation of the genotoxic and acute toxic effects of the drugs under realistic exposure conditions. It mimics the physiological conditions in the respiratory tract and eliminates the interactions of potential constituents with culture media (Thorne and Adamson 2013). These experiments were conducted with the buccal cells (TR-146) and with the human cell line A-459 which is derived from lung fibroblasts (Lieber et al. 1976) and was used in earlier gaseous exposure experiments (Majeed et al. 2014; Tang et al. 2012).

## Materials and methods

### Chemicals

Low-melting-point agarose (LMPA) and normal-melting-point agarose (NMPA) were obtained from Gibco (Paisley, UK). Inorganic salts, 2-aminoanthracene (2-AA), dimethyl sulfoxide (DMSO), propidium iodide, hydrogen peroxide, RPMI 1640, Triton X-100, Trizma base, trypan blue, Histopaque 1077, fetal calf serum (FCS), bovine serum albumin (BSA), 2-nitrofluorene (2-NF), 4-nitroquinoline-*N*-oxide (4-NQO), mitomycin C, cytochalasin-B, Dulbecco’s phosphate-buffered saline (DPBS), Dulbecco’s modified Eagle’s medium (DMEM), l-glutamine and sodium pyruvate were purchased from Sigma-Aldrich (Steinheim, Germany). Phytohemagglutinin (PHA) was ordered from Remel Inc., (Lenexa, USA). Aroclor™ 1254-induced rat liver S9 was purchased from Trinova Biochem GmbH (Giessen, Germany). Trypsin–EDTA was obtained from Life Technologies (Karlsruhe, Germany).

### Test compounds

The synthetic cannabinoids (4-methoxyphenyl)(1-pentyl-1*H*-indol-3-yl)methanone, (RCS-4, CAS 1345966-78-0; C_21_H_23_NO_2_) and [1-(5-fluoropentyl)-1*H*-indol-3-yl](2,2,3,3-tetramethyl-cyclopropyl)methanone (XLR-11, CAS 1364933-54-9; C_21_H_28_FNO) were provided as crystalline solids (purity ≥98 %) by the State Bureau of Criminal investigation Baden-Württemberg and the Finnish customs, respectively. Stock solutions were prepared in DMSO and stored at −20 °C.

### *Salmonella*/microsome assays

The compounds were tested in Ames microplate format (MPF) assays according to the instructions of the manufacturer [Ames MPF™, Xenometrix AG, Allschwil, Switzerland; see also Fluckiger-Isler and Kamber (2012)]. This procedure differs from the plate incorporation protocol as it is performed in liquid medium with 384-well microplates and it was used as lower amounts of the test compounds that are required than in plate incorporation experiments. The experiments were conducted with the *S. typhimurium* strains TA98 (*his*D3052, *rfa*, Δ*bio*, *uvr*B, pKM101) and TA100 (*his*G46, *rfa*, Δ*bio*, *uvr*B, pKM101). TA100 detects base pair substitutions while TA98 is sensitive towards mutagens which cause frameshifts (Maron and Ames 1983).

Different concentrations of the drugs (0.01, 0.1 and 1.0 mM) were tested in the presence and absence of metabolic activation mix (S9). 4-Nitroquinoline-*N*-oxide (4-NQO, 0.1 µg/mL) and 2-nitrofluorene (2-NF, 0.4 µg/mL) were used as positive controls in assays without metabolic activation. 2-Aminoanthracene (2-AA, 5.0 µg/mL) was used as positive control in all experiments with S-9 mix. Stock solutions of the test compounds were prepared with DMSO which was also added to control cultures according to the instructions of the manufacturer (Ames MPF™, Xenometrix AG, Allschwil, Switzerland).

Mutagenic effects were determined by measuring changes of the color of the wells from purple to yellow. The number of wells which contained his^+^ revertants was counted and compared with the solvent control. Each dose was tested in triplicate.

### Collection of lymphocytes

Peripheral blood samples were collected from three healthy, non-smoking male volunteers without any history of recent disease or exposure to toxic chemical agents. The samples were collected by venipuncture in heparinized tubes (BD, Heidelberg, Germany). Blood samples were collected for comet and MN assays on separate days from the same individuals.

For comet assays, blood samples from three donors were centrifuged (650*g*, 10 min, 4 °C) immediately after collection. Subsequently, the plasma was removed, the cell suspensions were diluted with RPMI 1640, and the lymphocytes were isolated by gradient centrifugation (800*g*, 16 °C min, 16 °C) with Histopaque 1077 in Accuspin tubes (Sigma-Aldrich, Steinheim, Germany). The cells were collected and washed twice in RPMI 1640 (332*g*, 10 min 16 °C), suspended in freezing medium (Biofreeze, Biochrom AG, Berlin, Germany) and stored at −80 °C.

Blood samples for cytokinesis-block micronucleus (CBMN) assays were collected from the same volunteers. The isolation of lymphocytes was performed as described by Fenech (2007). The cells were cultivated under standard conditions (37 °C, humidified atmosphere, 5 % CO_2_) in RPMI 1640 medium supplemented with 10 % FCS.

### Cultivation of human cell lines (TR-146 and A-549)

The human cell line TR-146 is derived from buccal epithelial tissue (Rupniak et al. 1985). The cells were obtained from J.G. Rheinwald (Dermatology Institute of Boston, MA USA). They were cultured under standard conditions (37 °C, humidified atmosphere, 5 % CO_2_) in DMEM which was supplemented with 10 % FCS. The cell line was stored in liquid nitrogen and was used for the experiments after recultivation at the fourth to the sixth passage from the stock cultures. The media were changed every 2–3 days; when the cultures had reached confluence, the cells were washed with Dulbecco’s phosphate-buffered saline (DPBS, Sigma-Aldrich, Steinheim, Germany), detached with Trypsin/EDTA, centrifuged and sub-cultured.

The human lung fibroblast line A-549 was obtained from the American Type Culture Collection (ATCC, Manassas, USA). The cells were used as an in vitro model for type II pulmonary epithelial cells and cultivated in RPMI 1640 medium (low glucose, with l-glutamine), supplemented with 10 % FCS and U/mL penicillin/streptomycin (Invitrogen, Darmstadt, Germany) under humidified conditions (5 % CO_2_; humidified atmosphere, 37 °C). Frozen human lung fibroblast cells were thawed and used for the experiments after the fourth to the sixth passage. Every 2–3 days, the medium was changed. At 85–90 % confluence, the cells were washed with Dulbecco’s PBS (Ca–Mg-free), then harvested using 0.25 % trypsin–EDTA and sub-cultured in T-25 cm^2^ flasks (Greiner Bio-One, Austria) or seeded on inserts.

### Single cell gel electrophoresis (SCGE) assays under standard conditions

SCGE assays were conducted under standard alkaline conditions (Tice et al. 2000). Frozen lymphocytes were thawed in a water bath (37 °C) and centrifuged (200 *g*, 5 min at 16 °C). Subsequently, the pellets were dissolved in RPMI 1640 and washed twice.

Aliquots of lymphocyte suspensions or of TR-146 cells (1 × 10^5^ cells) were treated (lymphocytes for 3 h, TR-146 for 24 h) with different concentrations (50–150 µM) of the synthetic cannabinoids. H_2_O_2_ (50 µM) was used as a positive control. Solutions of the drugs were prepared from deep-frozen stocks before each experiment and further dissolved in serum-free medium. After incubation in the dark (37 °C), shaking (250 rpm), the cells were washed twice with RPMI 1640 (containing 10 % FCS) and centrifuged (200*g*, 8 min, 16 °C). After centrifugation, the cytotoxic activities of the drugs were determined with the trypan blue exclusion test (Lindl and Bauer 1994); then the pellets were re-suspended in low-melting-point agarose (0.5 % LMPA). Subsequently, the cells were spread on pre-coated agarose slides (1.5 % NMPA) and lysed in the dark at 4 °C for at least 60 min. After 30 min unwinding under alkaline conditions (pH >13), electrophoresis was carried out for 30 min (300 mA, 1.0 V/cm, at 4 °C) and neutralization was performed twice for 8 min. Air-dried slides were stained with propidium iodide (10 µg/mL); subsequently the percentage of DNA in the tails was measured by use of a computer-aided image analysis system (Comet IV, Perceptive Instruments Ltd., Burry St. Edmunds´, UK). For each experimental point, three slides were prepared from each donor and 50 nuclei were evaluated randomly from each slide.

In addition to the dose–response experiments, assays were performed in which we investigated the impact of rat liver enzyme homogenate (S9) and bovine serum albumin (BSA) on comet formation. The incubation mixtures contained the indicator cells (1 × 10^5^ cells), solutions of the two drugs (final concentration 150 µM) and either PBS or fresh S-9 mix (final protein concentration 30 mg/mL) which was prepared according to the standard recipe of Maron and Ames (1983) or BSA solution (30 mg protein/mL). The mixtures were incubated for 3 h (37 °C; shaking 250 rpm); subsequently the cells were washed and processed as described above.

### Single cell gel electrophoresis (SCGE) assays with lesion-specific enzymes

To assess the impact of the drugs on formation of oxidatively damaged purines and pyrimidines, additional experiments with lesion-specific enzymes (formamidopyrimidine DNA glycosylase, FPG and endonuclease III, Endo III) were conducted according to the protocol of Collins and Dušinská (2002). The cells (TR-146) were exposed to the test compounds as described above. To establish the optimal amounts of the enzymes, calibration experiments were carried out according to the protocol of Collins et al. (1997) before the main experiments (results not shown).

After lysis, the slides were washed twice with enzyme reaction buffer (pH 8.0) for 8 min. Subsequently, the nuclei were treated either with 50 µL of the enzyme solutions or with the enzyme buffers. The incubation time for FPG was 30 min and for Endo III 45 min at 37 °C, respectively. After the treatment, electrophoresis was carried out under standard conditions (30 min, 300 mA, 1.0 V/cm, at 4 °C, pH >13). After lysis and electrophoresis, the slides were processed and evaluated as described above. For each experimental point, two cultures were made. From each culture, two slides were prepared and 50 cells were evaluated from each slide. The values which were obtained with the enzyme buffers were subtracted from the results which were obtained with the enzyme solutions.

### Cytokinesis-block micronucleus (CBMN) assays with human lymphocytes and TR-146 cells

Lymphocyte cultures were set up according to the recommendations of Fenech (2007) in agreement with the OECD guideline 487 (OECD 2010). Since human lymphocytes are more sensitive toward genotoxins during S, G2 and M phase than freshly collected cells which are in the G0 phase, mitogenic stimulation was performed with PHA before treatment of the cells with the test compounds.

Briefly, 1 × 10^6^ lymphocytes were added to 750 µL culture medium (RPMI 1640 containing 10 % FCS, 2.0 mM l-glutamine and 1.0 mM sodium pyruvate) and 10 µL of a PHA solution (30 µg/mL) to stimulate cell division. For each experimental point, two cultures were made; they were incubated at 37 °C in humidified atmosphere with 5 % CO_2_ with PHA for 44 h. Subsequently, the medium was replaced by fresh medium without FCS which contained different concentrations of the drugs (25.0, 50.0, 75.0, 100.0 and 150.0 µM). Treatment was performed for 3 h (37 °C, shaking 250 rpm in the dark). Cytochalasin-B was added to the cultures at a final concentration of 4.5 µg/mL to block cytokinesis after treatment with the drugs. Mitomycin C (1.0 µg/mL) was used as a positive control; serum-free medium with DMSO (1.0 %) was added to untreated cultures as a solvent control. After the treatment, the cells were washed twice with RPMI 1640 (332*g*, 10 min., 16 °C) and the pellets re-suspended in culture medium. The cells were harvested after 72 h. Slides were made with the cyto-centrifugation method (Fenech 2007); after drying, they were fixed and stained with Diff Quick (Dade Behring, Deerfield, IL, USA).

The protocol for experiments with TR-146 is described in detail in the paper of Koller et al. (2014). Briefly, the cells were seeded in 6-well plates and allowed to attach overnight. Subsequently, the medium was removed, and the cells were washed with DPBS; then they were treated with different concentrations of the test compounds in serum-free medium for 24 h. Mitomycin C (1.0 µg/mL) was used as a positive control; medium with DMSO (1 %) was used in the control cultures. After exposure of the cells to the drugs and washing with PBS, they were incubated with cytochalasin-B (3.0 µg/mL) and DMEM (with 10 % FCS) for another 24 h. Subsequently, the cells were washed, trypsinized and processed as described above.

From each culture, 2000 cells were evaluated. Different endpoints were scored, namely mono-nucleated, binucleated (BN) and multi-nucleated cells as well as the rates of binucleated cells with MN (BN–MN), the total number MN in binucleated cells (MNi), nuclear buds (Nbuds) and nucleoplasmatic bridges (NPBs). The cytokinesis-block proliferation indices (CBPI) were calculated with 500 cells according to the formula CBPI = [M1 + 2M2 +3(M3 + M4)]/N (N is the total number of scored cells, M1 − M4 refers to the number of cells with one to four nuclei). The toxicity of the compounds was indirectly assessed by the assumption that a CBPI of 1.0 corresponds to 100 % cytotoxicity. Five concentrations of each drug were used to determine the CBPI values. In agreement with OECD guideline 487 (OECD 2010), only doses were analyzed in regard to formation of MN and other nuclear anomalies which caused less than 60 % cytotoxicity.

### Determination of acute toxic and genotoxic properties of the drugs by use of air–liquid interface exposure system

The experiments were conducted with the VITROCELL^®^ cultivation and exposure system (12 HT/HT CF modules Waldkirch, Germany) with 6 cell culture inserts which is based on the cultivation of cells on porous membrane (Majeed et al. 2014). The system has separate exposure modules, each positioning three inserts which were used for parallel exposures to controls and test compounds. The aerosol is humidified up to 80 % under controlled temperature. The cells are nourished with serum-free medium from the basal surface, while being exposed to the aerosolized test compound in air–liquid interface (ALI) from the top that was flowing perpendicular onto the surface with constant air flow (5 mL/min).

The experiments were performed as described in an article of Weber et al. (2013) with slight modifications. The indicator cells (TR-146 and A-549, 1x10^5^ cells per insert) were transferred and grown in special Transwell^®^ inserts (ThinCerts™ Cell Culture Inserts, Greiner Bio-One, Frickenhausen, Germany) for 24 h before each experiment. Before the exposure, the media were removed from the apical surface of the cultures and transferred in sterile environment into the VITROCELL^®^ exposure system.

The cannabinoids (5 and 20 mg of each drug) were vaporized by a commercial vaporizer (Vaporizer-BLK-Titan, Near Dark GmbH, Hennef, Germany). Briefly, the drugs were placed inside the heating chamber of the apparatus and the temperature set on 220 °C to vaporize the chemicals. The cells were exposed to the vaporized drugs for 10 min (flow rate 5 mL/min/well) on the air–liquid interfaces via trumpets raised 0.5 cm above the cell layers. Flow and vacuum rates within the system were set using mass flow meters (Analyt-MTC GmbH, Mülheim, Germany). For each experimental point, two cultures were exposed from each culture and three slides were made. The experiments were conducted in duplicate.

After the treatment, the inserts were removed and transferred back to regular 12-well plates. Complete medium with 10 % FBS was added to both (the apical and the basal) surfaces. Subsequently, the cells were cultivated in an incubator, and then, the cultures were trypsinized and the cells analyzed in SCGE assays as described above. The cytotoxic effects of the drugs were monitored in all experiments with the trypan blue exclusion test (Lindl and Bauer 1994).

### Determination of concentrations of SCs in two cell lines

Quantification of the synthetic cannabinoids in cells was done using a LC/ESI–MS/MS system consisted of a QTrap^®^ 4000 mass spectrometer fitted with a TurbolonSpray interface (AB Sciex, Darmstadt, Germany), and a Shimadzu Prominence HPLC system (Duisburg, Germany) was used. Chromatographic separation was achieved using the conditions of Huppertz et al. (2014) with a Kinetex^®^ 2.6 u C18 100 Ǻ, 100 × 2.1 mm column (Phenomenex Ltd., Aschaffenburg, Germany) with a matching guard column and gradient elution with a flow rate of 0.5 mL/min (Huppertz et al. 2014).

The scheduled multiple reaction monitoring (sMRM) method contained two transitions for XLR-11, XLR-11 Isomer and RCS-4 and one transition for the ISTD. The MRM transitions were only recorded in a time window of ±50s around the expected retention time. For all compounds, the declustering potential (DP), the entrance potential (EP), the collision energy (CE) as well as the cell exit potential (CXP) were optimized. The mass spectrometer was operated in positive ionization mode. The method used was fully validated according to the guidelines of GTFCh; LODs are 0.04 ng/mL for RCS-4 and 0.03 ng/mL for XLR-11 and its isomer. Quantification was done using a 5-point calibration in the range of 0.1–1.25 ng/mL. After exposure of the cells (1 × 10^5^/insert) grown on the inserts, they were sonicated with 1000 µL MeOH, and subsequently, concentrations of the drugs were determined.

### Statistical analyses

All results were analyzed with GraphPad Prism 5 software system (LaJolla, CA, US). The data from the bacterial tests, from the SCGE experiments and from the MN assays are presented as mean ± SD.

The results of CBMN assays were analyzed with the Kruskal–Wallis test followed by Dunn’s test. The findings of SCGE assays (under standard conditions and after treatment with lesion-specific enzymes) were analyzed by one-way ANOVA followed by Dunnett’s multiple-comparisons test. The Student’s *t* test was used to analyze the data after liquid exposure to the drugs. Differences were considered as significant, when the *p* values were ≤0.05.

Results from Ames MPF assays were evaluated according to Fluckiger-Isler and Kamber (2012). Briefly, the mean numbers of positive (yellow) wells per dose were calculated from triplicates, and the fold increase above the baseline values was determined for each dose (for further details, see Fluckiger-Isler and Kamber 2012). A ≥2-fold increase compared to the baseline (control) value was considered as a positive result.

## Results

### *Salmonella*/microsome assays

The results of representative bacterial mutagenicity tests are summarized in Table 1. It can be seen that negative results were obtained with both drugs under all experimental conditions, while clear effects were found with the positive controls.

**Table 1:** Results of gene mutation assays obtained with the bacterial indicator strain, *Salmonella typhimurium* TA98 and TA100 in the presence and absence of metabolic activation mix

Compounds	Concentration	TA98 − S9	TA98 + S9	TA100 − S9	TA100 + S9
	mM	Mean ± SD	Mean ± SD	Mean ± SD	Mean ± SD
Neg. Cont.	0.00	0.7 ± 0.6	1.3 ± 1.2	3.7 ± 0.6	3.7 ± 1.5
RCS-4	0.01	0.3 ± 0.6	0.7 ± 1.2	3.7 ± 1.2	4.0 ± 3.5
	0.10	0.3 ± 0.6	3.0 ± 1.7	4.7 ± 0.6	4.0 ± 2.6
	1.00	1.0 ± 1.7	1.7 ± 0.6	2.7 ± 0.6	7.6 ± 1.2
XLR-11	0.01	0.3 ± 0.6	2.3 ± 1.5	5.3 ± 2.3	6.3 ± 2.9
	0.10	0.3 ± 0.6	1.7 ± 1.2	5.0 ± 2.1	6.7 ± 1.2
	1.00	1.0 ± 1.0	0.7 ± 1.2	3.3 ± 1.5	6.0 ± 2.0
Pos. Cont.^a^		38.3 ± 1.2*	48.0 ± 0.0*	48.0 ± 0.0*	36.0 ± 1.0*

The *Salmonella typhimurium* strains TA100 and TA98 were exposed to different concentrations of the SCs in the presence and absence of metabolic activation mix (rat liver S9) as described in materials and methods. The results were considered positive when the response was a ≥2-fold increase over that of the baseline. Numbers indicate means ± SDs of results obtained in three parallel experiments; asterisks indicate values which are significantly different from those found in the respective controls

* Significant differences from the controls cultures (*p* < 0.05)

^a^Positive controls without S9: 4-NQO (0.1 µg/mL) and 2-NF (0.4 µg/mL); with S9: 2-AA (5.0 µg/mL); DMSO was added to the negative controls

### Single cell gel electrophoresis experiments (standard conditions)

**Fig. 2:**
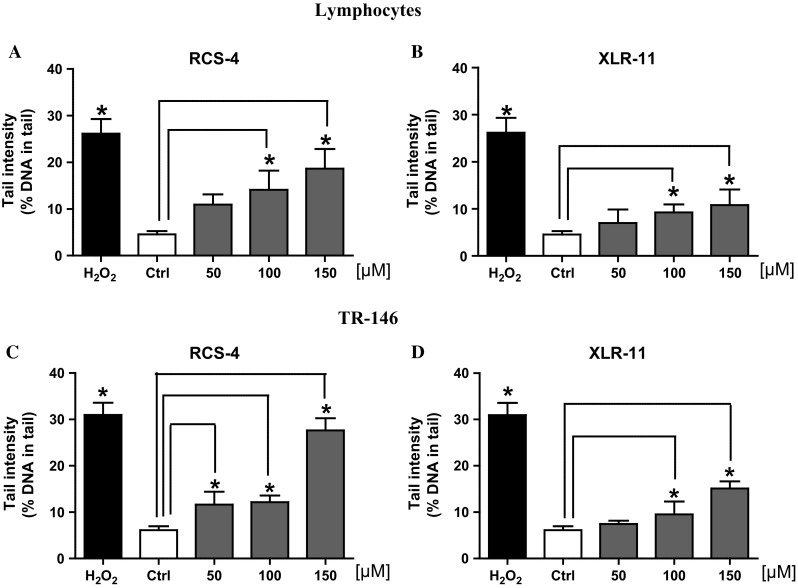
Impact of treatment of lymphocytes (**a**, **b**) and of human-derived buccal cells (TR-146; **c**, **d**) with RCS-4 and XLR-11 on comet formation. Lymphocytes were treated for 3 h, TR-146 cells for 24 h. *Bars* indicate mean ± SD of results obtained with three parallel cultures per experimental point in a representative experiment (from each culture, 2 slides were made and 50 cells were evaluated per slide). *Stars* indicate statistical significance (*p* ≤ 0.05, ANOVA)

Figure 2 depicts the results which were obtained with the drugs in the SCGE experiments with lymphocytes (Fig. 2a, b) and with TR-146 cells (Fig. 2c, d). With both SCs, dose-dependent induction of DNA migration was found in both cell lines. The doses which were required to cause significant effects were in most experimental series ≥100 µM; with RSC-4, a clear effect was seen in the buccal-derived cells already with 50 µM. The positive control (H_2_O_2_, 50 µM) induced in all experiments significant induction of DNA migration.

The cytotoxic effects of the drugs were monitored with the trypan blue exclusion test (Lindl and Bauer 1994). The ratio of “blue” cells (with membrane damage) was in all experiments <20 %; it is known that higher values may cause misleading results in SCGE experiments (Henderson et al. 1998).

### SCGE experiments with lesion-specific enzymes

**Fig. 3:**
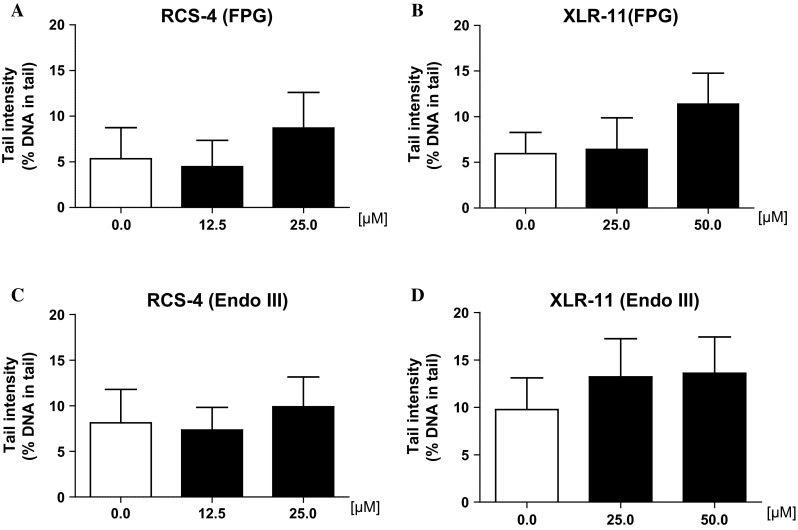
Impact of treatment of human-derived buccal cells (line TR-146) with synthetic cannabinoids on formation of oxidatively damaged purines (FPG-sensitive sites, **a**, **b**) and pyrimidines (Endo III sensitive sites, **c**, **d**). The cells were exposed to the drugs for 24 h. Subsequently, the nuclei were isolated and treated with enzymes or with the buffers only as described in the “Materials and methods” section. Subsequently, DNA migration was monitored. *Bars* indicate means ± SDs of results obtained with two cultures per experimental point in a representative experiment (values which were obtained with the enzyme buffers were subtracted from values which were obtained with the enzymes). From each culture, two slides were made and 50 cells were evaluated per slide

Several investigations indicate that SCs cause inflammations which may lead to release of reactive oxygen species [for further details, see (Bileck et al. 2015; Jean-Gilles et al. 2015)]. Therefore, we used a modified protocol of the SCGE technique to monitor formation of oxidatively damaged purines and pyrimidines. The results of these experiments are summarized in Fig. 3a–c. It can be seen that no evidence for increased formation of oxidized DNA bases was detected in the present experiments.

### SCGE experiments with liver S9 mix and bovine serum albumin

**Fig. 4:**
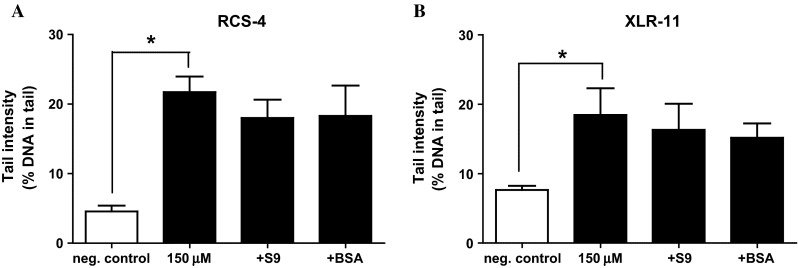
**a, b** Impact of rat liver S9 and bovine serum albumin (BSA) on SC-induced DNA migration in human lymphocytes. The cells were treated with solutions of the drugs (150 µM RCS-4 or XLR-11) with and without S9 mix or BSA (final protein concentration 0.3 mg/mL). After treatment of the cells, DNA migration was determined in SCGE experiments under standard condition. *Bars* represent means ± SDs of results obtained with tree cultures in parallel. From each culture, two slides were made and 50 cells were analyzed for comet formation per slide

To find out whether addition of phase I enzymes which are represented in liver-derived homogenate (S9) leads to activation of the drugs, further experiments were carried out. The results are depicted in Fig. 4a, b. It can be seen that the extent of comet formation caused by both drugs was reduced in the presence of the activation mix by 16 and 18 %, respectively. A similar effect was seen when the enzyme mix was replaced by BSA solution (which contained an identical amount of protein as the activation mix).

### Induction of acute toxic and genotoxic effects after vaporized exposure

The results of experiments in which the indicator cells were exposed in an air–liquid interface system are summarized in Table 2. Both SCs caused significant induction of DNA migration in the two indicator cell lines. Cytotoxic effects were in all experimental series below 20 % (columns 3 and 4). RCS-4 was in both indicator cell lines a more potent genotoxin, i.e., the extent of comet formation (expressed as % DNA in the tail) was under identical conditions approximately twofold higher with the benzoyl analog than with XLR-11.

**Table 2:** Impact of vaporized exposure on the DNA stability in human-derived lung fibroblasts (A-549) and buccal (TR-146) cells

Compounds	Dose (mg)	HPLC analyses (ng/mL)^a^		Cytotoxic activity (% cells of control)^c^		DNA migration (% DNA in tail)	
		A-549	TR-146	A-549	TR-146	A-549	TR-146
RCS-4	0.0	0.0	0.0	96.17 ± 5.46	97.87 ± 2.12	2.22 ± 0.75	2.03 ± 0.54
	5.0	n.d.^b^	1358.0	86.33 ± 3.81	84.93 ± 2.87	8.10 ± 2.91*	6.98 ± 4.21*
	0.0	0.0	0.0	94.67 ± 4.93	83.33 ± 1.53	3.72 ± 0.78	3.55 ± 1.09
	20.0	n.d.^b^	2987.0	81.50 ± 2.27	82.80 ± 2.52	10.1 ± 3.20*	7.04 ± 1.89*
XLR-11	0.0	0.0	0.0	95.50 ± 3.42	97.87 ± 2.12	1.76 ± 0.71	1.63 ± 0.47
	5.0	2010.0	1847.0	89.33 ± 9.35	87.60 ± 2.30	3.79 ± 1.07*	4.94 ± 2.03*
	0.0	0.0	0.0	95.60 ± 3.53	91.00 ± 6.25	2.60 ± 1.01	2.44 ± 0.90
	20.0	3030.0	4390.0	81.83 ± 3.54	84.47 ± 4.15	5.71 ± 1.42*	4.68 ± 1.24*

Numbers indicate means ± SDs of data obtained with three parallel cultures per experimental point in a representative experiment. From each culture, 3 slides were made and 50 cells were evaluated per slide

* Significant differences from the corresponding control (unexposed) cultures (Student’s *t* test, *p* ≤ 0.05). In each experiment, positive controls were included; i.e., cells were exposed for 5 min on ice H_2_O_2_ (50 µM); in all these experiments, significant induction of DNA migration was observed (data not shown)

^a^Values indicate means of four measurements. 10^4^ cells per insert were sonicated with 1000 µL MeOH; subsequently, the concentrations of the drug were determined (see “Materials and methods” section)

^b^ *n.d.* not determined

^c^Cytotoxicity was evaluated by use of trypan blue exclusion method. Numbers (%) indicate the ratio of cells with clear cytoplasm (cells without membrane damage) to total number of evaluated cells

### Impact of drugs on the induction of micronuclei and other nuclear anomalies

To find out whether treatment of human cells leads to formation of MN, which reflect structural and numerical chromosomal aberrations, cytome experiments were conducted. The results with lymphocytes and with TR-146 cells are summarized in Table 3.

**Table 3:** Impact of different synthetic cannabinoids on cytokinesis-block proliferation indices and on the rates of various nuclear aberrations in human mitogen-stimulated lymphocytes and in TR-146 cells

Compounds	Concentration	CBPI	CT	BN–MNi^a^	MNi^b^	Nbuds	NPBs
	µM	Mean ± SD	(%)	Mean (‰) ± SD	Mean (‰) ± SD	Mean (‰) ± SD	Mean (‰) ± SD
*Peripheral human lymphocytes*							
RCS-4	0	2.02 ± 0.14	0.0	4.14 ± 0.52	4.25 ± 0.49	2.96 ± 1.55	1.67 ± 0.75
	25	1.79 ± 0.15	22.6	6.45 ± 1.24	6.92 ± 0.96	3.73 ± 1.36	2.72 ± 1.24
	50	1.52 ± 0.22*	49.0	8.01 ± 0.7*	8.46 ± 1.12*	3.51 ± 1.05	2.98 ± 1.37
	75	1.25 ± 0.21*	75.5	n.e.	n.e	n.e.	n.e.
	100	1.19 ± 0.17*	81.4	n.e.	n.e	n.e.	n.e.
	150	1.07 ± 0.11*	80.4	n.e.	n.e	n.e.	n.e.
XLR-11	25	1.86 ± 0.20	16.7	7.03 ± 1.76	7.26 ± 1.86	4.31 ± 1.95	1.81 ± 1.00
	50	1.69 ± 0.22*	32.4	6.04 ± 2.50	6.16 ± 2.70	3.13 ± 0.77	2.45 ± 0.88
	75	1.59 ± 0.14*	43.1	8.54 ± 1.02	8.78 ± 1.02	4.48 ± 1.02	2.77 ± 0.66
	100	1.42 ± 0.13*	58.8	14.51 ± 4.40*	16.00 ± 5.71*	9.08 ± 3.04*	6.47 ± 3.02*
	150	1.07 ± 0.05*	93.1	n.e.	n.e.	n.e.	n.e.
Pos. Cont.	1 µg/mL	1.73 ± 0.16		48.36 ± 9.37*	50.62 ± 10.10*	12.44 ± 5.91*	2.68 ± 1.16
*Buccal-derived human cells (TR*-*146)*							
RCS-4	0	2.35 ± 0.44		1.39 ± 0.40	3.44 ± 0.34	1.34 ± 0.14	1.32 ± 0.34
	25	1.90 ± 0.75	33.3	3.46 ± 0.91	5.09 ± 1.09	3.92 ± 0.89	1.89 ± 0.97
	50	1.86 ± 0.32	36.3	4.67 ± 1.04	6.02 ± 1.02	3.35 ± 0.76	1.79 ± 0.50
	75	1.80 ± 0.25	40.7	6.89 ± 1.10*	7.99 ± 0.90*	3.92 ± 1.02	1.70 ± 0.40
	100	1.35 ± 0.86*	74.1	n. e.	n. e	n. e.	n. e
	150	1.21 ± 0.45*	84.4	n. e.	n. e	n. e.	n. e
XLR-11	25	1.99 ± 0.67	26.7	3.45 ± 0.97	5.20 ± 1.14	2.36 ± 0.89	1.12 ± 0.34
	50	1.78 ± 0.14	42.2	4.49 ± 0.52	6.09 ± 1.00	2.42 ± 0.09	1.42 ± 0.88
	75	1.75 ± 0.43*	44.4	6.46 ± 0.48*	7.08 ± 1.34*	2.99 ± 0.90	1.40 ± 0.36
	100	1.51 ± 0.54*	62.2	8.98 ± 1.23*	10.08 ± 2.10*	2.32 ± 0.93	1.76 ± 0.78
	150	1.20 ± 0.13*	85.2	n. e.	n. e.	n. e.	n. e.
Pos. Cont.	1 µg/mL	1.68 ± 0.09		45.21 ± 5.20*	53.21 ± 7.60*	10.34 ± 3.20*	2.05 ± 1.06

Human mitogen-stimulated lymphocytes from three individuals were treated with different concentrations of the test compounds for 3 h. TR-146 cells were exposed to different concentrations of the drugs for 24 h. Numbers represent results (means ± SDs) obtained with duplicate cultures from four donors. Per experimental point, two slides were prepared and 1000 cells were evaluated from each slide

*n.e.* not evaluated due to inhibition of cell division

Please exchange C^+^ to Pos. Cont. mitomycin C (1.0 μg/ml)

*CBPI* cytokinesis-block proliferation indices, *CT* cytostasis (%); *BN*–*MNi* binucleated cells with micronuclei, *MNi* total number of micronuclei, *Nbuds* nuclear buds, *NPBs* nucleoplasmic bridges, *0* solvent control (DMSO), *C* ^+^ mitomycin C (1.0 µg/mL)

* Significant differences from negative control (Kruskal–Wallis test followed by Dunns’s test, *p* ≤ 0.05)

^a^Number of binucleated cell with micronuclei

^b^Total number of MN

Both compounds caused in both cell types induction of MNi. A clear effect was found in the lymphocytes with XLR-11 with concentrations ≥100 µM, while in buccal-derived cells significant effects were detected already with a lower concentration (75 µM). Also RCS-4 caused a positive result in both types of indicator cells. A clear increase in the MN rates was detected in the lymphocytes with 50 µM; higher doses could be not evaluated due to inhibition of cell division.

With XLR-11 additionally a significant increase in other nuclear anomalies (Nbuds and NPBs) was observed in the blood cells and also in the buccal cells at high doses (100 and 150 µM, respectively). The rates of these anomalies were also elevated in both cell types as a consequence of treatment with RCS-4, but these effects were only moderate and did not reach significance.

## Discussion

Three different genotoxicity systems were used in the present study to investigate the genotoxic properties of XLR-11, one of the most widely consumed SCs and, of the benzoyl indole RCS-4. Furthermore, attempts were made to characterize the molecular mechanisms which lead to damage of the genetic material and to find out whether adverse effects can be expected in drug users under realistic exposure conditions.

The negative results which were obtained in the *Salmonella*/microsome assays indicate that the drugs do not induce gene mutations. These findings were not unexpected; also with other indole SCs including UR-144, which is structurally related to XLR-11 (XLR-11 is the fluorinated form of UR-144) (Mohr et al. 2014), consistently negative results were obtained in earlier bacterial mutagenicity tests (Koller et al. 2015); also the natural cannabinoid Δ^9^-THC does not cause induction of his^+^ revertants in *Salmonella* tester strains (NTP 1996). SCs with benzoyl structure like RCS-4 have not been studied before in microbial mutagenicity assays according to our knowledge, and the present findings show that the compound is devoid of activity under all experimental conditions.

On the contrary, clear evidence for induction of DNA damage was found in SCGE experiments which reflect single- and double-strand breaks as well as apurinic sites (Tice et al. 2000) in human lymphocytes and also in the buccal-derived cells (Fig. 2). In this context, it is notable that we observed induction of comet formation by other SCs in earlier experiments; for example, the cyclohexylphenol CP-47,497-C8 caused significant induction of DNA migration in lymphocytes and in TR-146 cells (Bileck et al. 2015; Koller et al. 2014). Also with UR-144 and alkylindazoles (AM-2201-IC and 5F-AKB-48), positive findings were obtained in human blood cells (Koller et al. 2015) and representatives of naphthoylindoles (JWH-122 and JWH-073) induced DNA migration in buccal-derived cells (Koller et al. 2013). The concentrations of the drugs which were required to cause significant effects were in most cases in the range between 50 and 100 µM; only with one compound (CP-47,497-C8), significant induction of DNA migration was seen at a substantially lower dose (10 µM). Also with Δ^9^-THC, induction of comets has been reported, for example, in the human-derived buccal cell line which was also used in the present experiments (Koller et al. 2013) and also in an environmental study with mussels (Parolini and Binelli 2014).

It is not known in general whether positive results in SCGE experiments lead to persisting alterations of the genetic material. In order to find out whether the two drugs cause damage at the chromosomal level which is associated with adverse health effects, we conducted cytome assays in which we studied the formation of MNi which are a consequence of structural and numerical aberrations (Norppa and Falck 2003) as well as other nuclear anomalies. As described in the results sections (Table 2), positive results were obtained with both drugs in the buccal cell line (TR-146) and also in the lymphocytes. Also the rates of other nuclear anomalies namely Nbuds and NPBs lower elevated after treatment of the lymphocytes with XLR-11. It is known that these anomalies reflect genetic instability; i.e., NPBs are formed as a consequence of dicentric chromosomes while Nbuds reflect gene amplification events (Fenech et al. 2011). The biological consequences of the formation of these anomalies are not known at present; in the case of MNi, it is notable and it was shown by Bonassi and co-workers that increased rates of MN in peripheral lymphocytes of humans are associated with elevated cancer risks (Bonassi et al. 2007, 2011).

The results of the present experiments allow to draw some conclusions in regard to the molecular mechanisms which lead to damage of the genetic material. The negative results in the bacterial assays indicate that the drugs do not form bulky DNA adducts as other groups of genotoxic carcinogens (e.g., aflatoxins, polycyclic aromatic hydrocarbons or heterocyclic amines) which cause gene mutations and lead to formation of his^+^ revertants. Furthermore, it can be also excluded that the induction of the comets which was observed in the SCGE experiments is causally related to formation of oxidatively damaged purines and pyrimidines. As shown in Fig. 3a–d, negative results were consistently obtained in experiments with lesion-specific enzymes (FPG, Endo III) which enable the detection of oxidized DNA bases (Collins 2014). In this context, it is notable that it was found in earlier investigations that the natural cannabinoid Δ^9^-THC induces oxidative damage in eukaryotic cells (Juknat et al. 2013; Parolini and Binelli 2014; Sarafian et al. 1999); furthermore, SCs were reported to cause induction of pro-inflammatory cytokines which may lead to release of reactive oxygen species (Bileck et al. 2015; Jean-Gilles et al. 2015).

Many genotoxic carcinogens require metabolic activation via phase I enzymes. To mimic these metabolic processes which lead to formation of DNA-reactive metabolites in vivo, liver enzyme homogenate (S9 mix) is added in routine mutagenicity assays (OECD 1997, 2010). As shown in Fig. 4a, b, we found that addition of the enzyme homogenate leads to a moderate (not significant) decrease in the genotoxic properties of both drugs. A similar effect was seen when BSA was added to the incubation mixtures. These findings show that enzymes which are contained in liver homogenates do not convert these drugs to DNA-reactive intermediates. The observation of a reduction in their genotoxic activity which was seen with the enzyme mix and with pure proteins may be indicative of detoxification via (non-enzymatic) protein binding. Similar, but more pronounced effects were seen in previous experiments with the SC, CP-47,497-C8 (Koller et al. 2014) and also with representatives of other groups of directly acting mutagens for example with isothiocyanates and alkylating agents (Kassie and Knasmuller 2000). It is possible that these detoxification reactions play also a role in the human body in addition to metabolic processes which are catalyzed by enzymes (e.g., hydroxylation, carboxylation and glucuronidation) which were observed recently in in vitro experiments with XLR-11 (Wohlfarth et al. 2013).

The last part of this study concerned the question whether the drugs cause DNA damage under realistic exposure conditions. Only for few SCs (including RCS-4), data on their concentrations in body fluids of humans after consumption of the drugs are available (Kneisel et al. 2013; Teske et al. 2010). The concentrations which were detected are 2–3 orders of magnitude lower than those which caused positive results in the present experiments. In order to assess whether induction of genetic damage occurs in users as a consequence of inhalation of the drugs, we conducted with both SCs experiments with an air–liquid interface system. As described above, the cells were grown on membranes and exposed to drugs under conditions which mimic the exposure of tissues in the respiratory tract. The amounts which were vaporized (5 and 20 mg) are contained in 1–2 typical SC containing cigarettes (Adamowicz et al. 2013). As described in the results section, we found with both SCs clear evidence for induction of DNA migration in both types of indicator cells.

Taken together, the results of the present study show that both drugs cause DNA instability in human-derived cells at the chromosomal level. It is known that instability of the genetic material plays a key role in the etiology of human cancer (Ferguson et al. 2015). Furthermore, it is also involved in neurodegenerative disorders (Madabhushi et al. 2014), infertility (Evgeni et al. 2015) and accelerated aging (Ermolaeva and Schumacher 2014). Therefore, the findings of our investigation indicate that adverse health effects may be caused in drug users as a consequence of the DNA-damaging properties of the drugs, and further investigations are warranted to clarify this important issue.

## Abbreviations

2-AA: 2-Aminoanthracene
BN–MN: Binucleated cells with micronucleus
CBMN assay: Cytokinesis-block micronucleus assay
CBPI: Cytokinesis-block proliferation index
CT: Cytostasis
2-NF: 2-Nitrofluorene
MN: Micronucleus
MNi: Micronuclei
Nbuds: Nuclear buds
4-NQO: 4-Nitroquinoline-*N*-oxide
NPBs: Nucleoplasmic bridges
PHA: Phytohemagglutinin
RCS-4: (4-Methoxyphenyl)(1-pentyl-1*H*-indol-3-yl)methanone
SCGE: Single cell gel electrophoresis
SCs: Synthetic cannabinoids
THC: Tetrahydrocannabinol
XLR-11: [1-(5-Fluoropentyl)-1H-indol-3-yl](2,2,3,3-tetramethylcyclo-propyl)methanone

## References

[CR1] Adamowicz P, Zuba D, Sekula K (2013). Analysis of UR-144 and its pyrolysis product in blood and their metabolites in urine. Forensic Sci Int.

[CR2] Ames BN, Gold LS (1997). The causes and prevention of cancer: gaining perspective. Environ Health Perspect.

[CR3] Ames BN, Shigenaga MK, Hagen TM (1993). Oxidants, antioxidants, and the degenerative diseases of aging. Proc Natl Acad Sci USA.

[CR4] Bileck A, Koller V, Ferk F et al (2015) Impact of a synthetic cannabinoid (CP-47,497-C8) on protein expression in human cells: evidence for induction of inflammations and DNA-damage. Arch Toxicol (**Published online 21 July 2015**) 10.1007/s00204-015-1569-7 26194647

[CR5] Bonassi S, Znaor A, Ceppi M (2007). An increased micronucleus frequency in peripheral blood lymphocytes predicts the risk of cancer in humans. Carcinogenesis.

[CR6] Bonassi S, El-Zein R, Bolognesi C, Fenech M (2011). Micronuclei frequency in peripheral blood lymphocytes and cancer risk: evidence from human studies. Mutagenesis.

[CR7] Collins AR (2014). Measuring oxidative damage to DNA and its repair with the comet assay. Biochim Biophys Acta.

[CR8] Collins A, Dušinská M, Armstrong D (2002). Oxidation of cellular DNA measured with the comet assay. Oxidative stress biomarkers and antioxidant protocols. Methods in molecular biology.

[CR9] Collins AR, Dobson VL, Dusinska M, Kennedy G, Stetina R (1997). The comet assay: what can it really tell us?. Mutat Res.

[CR10] Ermolaeva MA, Schumacher B (2014). Systemic DNA damage responses: organismal adaptations to genome instability. Trends Genet.

[CR11] Evgeni E, Lymberopoulos G, Gazouli M, Asimakopoulos B (2015). Conventional semen parameters and DNA fragmentation in relation to fertility status in a Greek population. Eur J Obstet Gynecol Reprod Biol.

[CR12] Fenech M (2007). Cytokinesis-block micronucleus cytome assay. Nat Protoc.

[CR13] Fenech M, Kirsch-Volders M, Natarajan AT (2011). Molecular mechanisms of micronucleus, nucleoplasmic bridge and nuclear bud formation in mammalian and human cells. Mutagenesis.

[CR14] Ferguson LR, Chen H, Collins AR, Connell M, Damia G, Dasgupta S, Malhotra M (2015). Genomic instability in human cancer: molecular insights and opportunities for therapeutic attack and prevention through diet and nutrition. Semin Cancer Biol.

[CR15] Fluckiger-Isler S, Kamber M (2012). Direct comparison of the Ames microplate format (MPF) test in liquid medium with the standard Ames pre-incubation assay on agar plates by use of equivocal to weakly positive test compounds. Mutat Res.

[CR16] Henderson L, Wolfreys A, Fedyk J, Bourner C, Windebank S (1998). The ability of the Comet assay to discriminate between genotoxins and cytotoxins. Mutagenesis.

[CR17] Huppertz LM, Kneisel S, Auwarter V, Kempf J (2014). A comprehensive library-based, automated screening procedure for 46 synthetic cannabinoids in serum employing liquid chromatography-quadrupole ion trap mass spectrometry with high-temperature electrospray ionization. J Mass Spectrom.

[CR18] Jean-Gilles L, Braitch M, Latif ML (2015). Effects of pro-inflammatory cytokines on cannabinoid CB1 and CB2 receptors in immune cells. Acta Physiol (Oxf).

[CR19] Juknat A, Pietr M, Kozela E (2013). Microarray and pathway analysis reveal distinct mechanisms underlying cannabinoid-mediated modulation of LPS-induced activation of BV-2 microglial cells. PLoS ONE.

[CR20] Kassie F, Knasmuller S (2000). Genotoxic effects of allyl isothiocyanate (AITC) and phenethyl isothiocyanate (PEITC). Chem Biol Interact.

[CR21] Kneisel S, Auwarter V, Kempf J (2013). Analysis of 30 synthetic cannabinoids in oral fluid using liquid chromatography-electrospray ionization tandem mass spectrometry. Drug Test Anal.

[CR22] Koller VJ, Zlabinger GJ, Auwarter V, Fuchs S, Knasmueller S (2013). Toxicological profiles of selected synthetic cannabinoids showing high binding affinities to the cannabinoid receptor subtype CB(1). Arch Toxicol.

[CR23] Koller VJ, Auwarter V, Grummt T, Moosmann B, Misik M, Knasmuller S (2014). Investigation of the in vitro toxicological properties of the synthetic cannabimimetic drug CP-47,497-C8. Toxicol Appl Pharmacol.

[CR24] Koller VJ, Ferk F, Al-Serori H (2015). Genotoxic properties of representatives of alkylindazoles and aminoalkyl-indoles which are consumed as synthetic cannabinoids. Food Chem Toxicol.

[CR25] Lieber M, Smith B, Szakal A, Nelson-Rees W, Todaro G (1976). A continuous tumor-cell line from a human lung carcinoma with properties of type II alveolar epithelial cells. Int J Cancer.

[CR26] Lindl T, Bauer J (1994). Zell- und Gewebekultur.

[CR27] Logan BK, Reinhold LE, Xu A, Diamond FX (2012). Identification of synthetic cannabinoids in herbal incense blends in the United States. J Forensic Sci.

[CR28] Madabhushi R, Pan L, Tsai LH (2014). DNA damage and its links to neurodegeneration. Neuron.

[CR29] Majeed S, Frentzel S, Wagner S (2014). Characterization of the Vitrocell(R) 24/48 in vitro aerosol exposure system using mainstream cigarette smoke. Chem Cent J.

[CR30] Maron DM, Ames BN (1983). Revised methods for the Salmonella mutagenicity test. Mutat Res.

[CR31] Mohr AL, Ofsa B, Keil AM, Simon JR, McMullin M, Logan BK (2014). Enzyme-linked immunosorbent assay (ELISA) for the detection of use of the synthetic cannabinoid agonists UR-144 and XLR-11 in human urine. J Anal Toxicol.

[CR32] Norppa H, Falck GC (2003). What do human micronuclei contain?. Mutagenesis.

[CR33] NTP (1996). National Toxicology Program—toxicology and carcinogenesis studies of 1-trans-delta(9)-tetrahydrocannabinol (CAS No. 1972-08-3) in F344 Rats and B6C3F1 Mice (Gavage Studies). Natl Toxicol Program Tech Rep Ser.

[CR34] OECD (1997) Test no. 471: bacterial reverse mutation test. OECD (Organisation for Economic Co-operation and Development) guidelines for the testing of chemicals, section 4. OECD Publishing, Paris. doi:10.1787/9789264071247-en

[CR35] OECD (2010) Test no. 487: in vitro mammalian cell micronucleus test. OECD (Organisation for Economic Co-operation and Development) guidelines for the testing of chemicals, section 4. OECD Publishing, Paris. doi:10.1787/9789264091016-en

[CR36] Parolini M, Binelli A (2014). Oxidative and genetic responses induced by Delta-9-tetrahydrocannabinol (Delta-9-THC) to Dreissena polymorpha. Sci Total Environ.

[CR37] Rupniak HT, Rowlatt C, Lane EB (1985). Characteristics of four new human cell lines derived from squamous cell carcinomas of the head and neck. J Natl Cancer Inst.

[CR38] Sarafian TA, Magallanes JA, Shau H, Tashkin D, Roth MD (1999). Oxidative stress produced by marijuana smoke. An adverse effect enhanced by cannabinoids. Am J Respir Cell Mol Biol.

[CR39] Seely KA, Patton AL, Moran CL (2013). Forensic investigation of K2, Spice, and “bath salt” commercial preparations: a three-year study of new designer drug products containing synthetic cannabinoid, stimulant, and hallucinogenic compounds. Forensic Sci Int.

[CR40] Spaderna M, Addy PH, D’Souza DC (2013). Spicing things up: synthetic cannabinoids. Psychopharmacology.

[CR41] Tang T, Gminski R, Könczöl M, Modest C, Armbruster B, Mersch-Sundermann V (2012). Investigations on cytotoxic and genotoxic effects of laser printer emissions in human epithelial A549 lung cells using an air/liquid exposure system. Environ Mol Mutagen.

[CR42] Teske J, Weller JP, Fieguth A, Rothamel T, Schulz Y, Troger HD (2010). Sensitive and rapid quantification of the cannabinoid receptor agonist naphthalen-1-yl-(1-pentylindol-3-yl)methanone (JWH-018) in human serum by liquid chromatography-tandem mass spectrometry. J Chromatogr, B: Anal Technol Biomed Life Sci.

[CR43] Thorne D, Adamson J (2013). A review of in vitro cigarette smoke exposure systems. Exp Toxicol Pathol.

[CR44] Tice RR, Agurell E, Anderson D (2000). Single cell gel/comet assay: guidelines for in vitro and in vivo genetic toxicology testing. Environ Mol Mutagen.

[CR45] Uchiyama N, Kawamura M, Kikura-Hanajiri R, Goda Y (2013). URB-754: a new class of designer drug and 12 synthetic cannabinoids detected in illegal products. Forensic Sci Int.

[CR46] Weber S, Hebestreit M, Wilms T, Conroy LL, Rodrigo G (2013). Comet assay and air-liquid interface exposure system: a new combination to evaluate genotoxic effects of cigarette whole smoke in human lung cell lines. Toxicol In Vitro.

[CR47] Wiley JL, Marusich JA, Lefever TW, Grabenauer M, Moore KN, Thomas BF (2013). Cannabinoids in disguise: Delta9-tetrahydrocannabinol-like effects of tetramethyl-cyclopropyl ketone indoles. Neuropharmacology.

[CR48] Wohlfarth A, Pang S, Zhu M (2013). First metabolic profile of XLR-11, a novel synthetic cannabinoid, obtained by using human hepatocytes and high-resolution mass spectrometry. Clin Chem.

